# Photovoice as a Method to Reduce the Stigma of Mental Illness Among Health Care Students

**DOI:** 10.1177/15248399211057152

**Published:** 2022-03-14

**Authors:** Gregory K. Tippin, K. Amanda Maranzan

**Affiliations:** 1 Lakehead University, Thunder Bay, Ontario, Canada

**Keywords:** mental health, health promotion, outcome evaluation, program planning and evaluation

## Abstract

Photovoice is theorized to influence those who interact with the photos and captions, and so it is important to examine and further understand this mechanism. This article seeks to further our understanding of this critical process—that is, what is the impact of the Photovoice Artist’s stories on the viewing audience? Herein we demonstrate how an incarnation of photovoice, digital storytelling, and photo elicitation impacted mental illness stigma among health sciences students. By focusing on application beyond the original exhibition, this article highlights how photovoice methods and aims overlap with best practices in stigma reduction, and its fit with multimodal anti-stigma interventions. Overall, this study contributes to addressing the question of how photovoice can be applied to achieve action for social change.

People with lived experience of a mental illness frequently encounter stigma from health care professionals such as dehumanization, being treated as a child, exclusion from decision-making, and pessimism about recovery (Henderson et al., 2014). There is also some literature to suggest that health care professionals hold more stigmatizing depictions/stereotypes of patients compared with the general public (Nordt et al., 2006). Health care providers make decisions that directly impact opportunities for people with mental illnesses (Corrigan, 2004), and personal stigma among health care providers has been linked to poorer quality of physical and mental health care for this group (Hatzenbuehler et al., 2013). Stigma toward people with mental illnesses also exists among health care students including those studying nursing (Bingham & O’Brien, 2018; Clement et al., 2012; Surgenor et al., 2005) and as future health care providers, student trainees are an important subgroup to reach for anti-stigma intervention.

## Photovoice and Stigma Reduction

The stigma literature is clear that contact is the most important component of strategies to reduce stigma (Corrigan et al., 2012). Contact-based anti-stigma interventions involve direct interactions with members of a stigmatized group (e.g., mental illness) and are based on the work of Allport (1954) who proposed that increased contact with a stigmatized group can reduce prejudice toward that group. Knowledge about the outgroup (i.e., people with mental illnesses), reducing anxiety about intergroup contact, and empathy/perspective-taking are three key mechanisms through which contact reduces stigma (Pettigrew & Tropp, 2008). Anti-stigma campaigns often use filmed contact (e.g., an individual with a mental illness describes their experiences) which has also been shown to reduce stigma (Clement et al., 2012).

Photovoice methods and aims overlap with several of the mechanisms of change underlying contact-based anti-stigma interventions: conveying knowledge about group experiences and perspectives, through group members’ own photos and words. Photovoice is frequently used to convey the experience of living as a member of a marginalized group (Fleming et al., 2009) in order to document and address social and health issues (Becker et al., 2014; Catalani & Minkler, 2010; Hergenrather et al., 2009; Perez et al., 2016; Russinova et al., 2018). As people with mental illnesses frequently experience discrimination and negative attitudes in various spheres (such as access to and receipt of healthcare; housing; employment; relationships), photovoice is a powerful method to document and communicate these experiences. Photovoice has explored the experience of mental illness (Fleming et al., 2009), the meaning of recovery for individuals with serious mental illnesses (Cabassa et al., 2013; Mizock et al., 2014), and university students’ experiences of mental illness stigma on campus (Wada et al., 2019). Common themes in stigma-focused photovoice works created by persons with serious mental illnesses included symbolic representations of stigma, negative stigma impacts, stigma coping strategies, personal stigma-related transformation, and educational messages for others (Russinova et al., 2018).

An understudied aspect of photovoice is the impact of photovoice photographer’s stories on the viewing audience, yet there is some evidence that photovoice can reduce public and personal stigma. Flanagan and colleagues (2016) found that primary care providers who viewed a photovoice intervention followed by discussion (“Recovery Speaks”) demonstrated decreased negative stereotypes, attributions of dangerousness, fear, and decreased desire for treatment coercion, segregation, and avoidance. Knaak and Patten (2013) found reduced audience stigma associated with viewing a documentary about individuals creating a photovoice presentation. Our research team was invited to assess outcomes associated with a photovoice project that was initiated and developed by the Canadian Mental Health Association–Thunder Bay branch. The photovoice project is an example of grassroots collaboration (i.e., a local program, working with people who have experience with mental illnesses), an approach that is consistent with recommendations emphasizing the need for collaboration with people with lived experience in anti-stigma interventions (Corrigan, 2014). The organization’s goal was to design an educational tool to help change attitudes about people living with mental illnesses, by conveying the experience of living with a mental illness, barriers and supports to recovery, and how others can help support recovery. We previously demonstrated that the photovoice presentation itself reduced stigma among undergraduate students (Tippin & Maranzan, 2019).

## Purpose

This article demonstrates the use of photovoice at the centre of a multimodal anti-stigma intervention delivered to nursing and kinesiology undergraduate students.

## Method

### Participants

Participants were 52 postsecondary students enrolled in nursing and kinesiology programs at a mid-sized Canadian university. The multimodal photovoice-based anti-stigma intervention occurred during a regularly scheduled course (6 nursing; 1 kinesiology course), and students could opt in to complete pre, post, and follow-up measures as part of this study. This project was approved by the institutional Research Ethics Board, and all students provided informed consent prior to taking part in the study.

### Photovoice-Based Intervention

The photovoice-based intervention was a 1.25-hour presentation, developed by the Canadian Mental Health Association–Thunder Bay Branch and local consumers of mental health services, featuring education, photovoice, and live contact with an individual with lived experience of mental illness (i.e., one of the Photovoice Artists). The photovoice video served as the centerpiece of the multimodal intervention, with the education and live contact components of the intervention tailored to the content of the photovoice video and structured to follow a fidelity checklist covering the intervention elements described herein. An educator first discussed concepts related to mental disorder labels, stigma, and recovery, and briefly described the rationale and implementation of the photovoice project. Next, the photovoice video was shown, and students were instructed to choose two photographs from the photovoice presentation that were personally salient for discussion following the viewing.

This photovoice video was created by people with lived experience of mental illness and previously described by Tippin and Maranzan (2019). To summarize, over a 9-month period, eight consumers of mental health services (described herein as the photovoice artists) were given disposable cameras and asked to take photographs that represented their experience of living with or recovering from a mental illness. The goal from the start of the project was to use the photovoice data in a manner that could be shared with various general audiences (e.g., public viewings; organizations), including the creation of voiceovers to further convey the experience depicted in the photos, all in an effort to reduce stigma. Ultimately, 30 photographic images were collaboratively chosen and organized by the group based on several themes: the experience of living with a mental illness, barriers to recovery, supports for recovery, and the overall experience of recovery. Voiceovers were recorded for each photo explaining its meaning to the photographer’s experience (photographers only chose from among the photos they took themselves), with an accompanying phototext derived from the voiceover. The end product was a 19-minute video, consisting of each photo accompanied by its associated voiceover and phototext. The photovoice presentation addressed a range of mental health problems (psychosis, depression, substance use, bipolar disorder, general mental illness) and emphasized recovery principles via the aforementioned themes.

After viewing the photovoice video, a Photovoice Artist led a discussion with the audience that included personal testimony of their experience with mental illness and recovery, engaged the audience in discussing salient photographs from the photovoice video, discussed attitudes and actions that support recovery, and provided opportunities for audience questions. Each presentation was rated for topic adherence using a fidelity checklist in order to ensure a minimum level of consistency in the intervention content across classes.

### Measures

Students completed measures that assessed demographic characteristics, response style (social desirability), and mental illness stigma. All stigma measures were administered online at three time points: 1 week pre-intervention, 1 week post-intervention, and at 1-month follow-up.

#### Demographic Information

We considered a number of student characteristics for descriptive purposes, including age, gender, ethnicity, university major, and years of university completed. These characteristics were chosen to describe and contextualize the student sample within the larger stigma literature where there is some evidence of associations between demographic characteristics (e.g., age; ethnicity) and stigma. There is also some literature to suggest that year of study is related to stigmatizing attitudes among nursing students, with first-year students holding more personal stigma (Surgenor et al., 2005) and so university major and year/level were important contextual variables. We also considered prior experience/familiarity with mental illnesses (measured with the Level of Contact Report; Holmes et al., 1999); 6% of the student sample reported having a mental illness or illnesses.

#### Social Desirability

The *Balanced Inventory of Desirable Responding* (Paulhus, 1991) was used as a proxy of the possible influence of social desirability. The Balanced Inventory of Desirable Responding contains 40 self-report items rated on a 7-point Likert-type scale, with higher scores indicative of self-deceptive enhancement (the tendency to respond honestly but to provide positively biased answers) and impression management (intentional attempts to endorse socially desirable responses).

#### Stigma

Mental illness stigma was measured with several commonly used self-report scales. The *Unpredictability-Incompetence* scale (UI; Angermeyer & Matschinger, 2004) measured the degree to which students perceive people with a mental illness as being unpredictable and incompetent in self-care and general demeanor. The *Dangerousness Scale* (DS; Link et al., 1987) measured students’ perceptions of the dangerousness of people with a mental illness, including stigmatizing cognitive and behavioural reactions. The *Social Distance Scale* (SDS; Link et al., 2004) measured stigmatizing behavioural reactions (i.e., discrimination) toward people with a mental illness. Finally, several subscales from the *Attribution Questionnaire* (AQ; Corrigan et al., 2002) were used to assess students’ stigmatizing and prosocial responses in areas that were not covered by the aforementioned measures. We used subscales to measure students’ perceptions of responsibility (of a person for their mental illness), and anger, fear, pity, and empathy toward people with a mental illness. On all measures, higher scores indicate a higher level of personal stigma.

## Results

A total of 52 students completed pre- and post-intervention measures. Data were examined for missing items, outliers, and normality. Mean substitution was used if a student did not answer 1 item from a given measure; otherwise pairwise deletion was used in relevant analyses. A logarithmic (base 10) transformation was used on the AQ-Fear subscale to reduce one univariate outlier and to improve normality.

### Stigma

A series of paired-samples *t* tests were used to compare students’ pre- and post-intervention scores on our stigma measures. Students reported a decrease in their perception of people with a mental illness as being unpredictable and incompetent, *t*(50) = 2.30, *p* < .05, decreased desire for social distance, *t*(50) = 5.30, *p* < .001, and decreased fear of people with a mental illness, *t*(50) = 2.91, *p* < .01. Benjamini and Hochberg’s (1995) false discovery rate was used to protect against type 1 error; all original *p* values remained significant and satisfied false discovery rate critical values set at 10%. Conservative effect size estimates were calculated following a formula from Dunlap et al. (1996), accounting for the correlation between participants’ pre-post scores on each measure in this single-group design. Cohen’s d (1988) estimates indicated small effects associated with these statistically significant changes (ranged from .28 to .44). The Balanced Inventory of Desirable Responding was not correlated with any of our stigma measures, suggesting that social desirability did not play a significant role in students’ responses.

### One-Month Follow-Up

To examine 1-month outcomes associated with the photovoice-based intervention, our stigma measures were re-administered 1 month later. One third of the original sample (36%) completed the follow-up measures, and this group did not differ in terms of demographics or any of our baseline measures. At 1-month follow-up, students continued to endorse significantly reduced desired social distance from people with a mental illness relative to pre-intervention scores, *t*(18) = 2.73, *p* < .01, and significantly increased pity (AQ-Pity) toward people with a mental illness at follow-up, *t*(18) = −5.17, *p* < .001 (notably, this was not significant immediately post-intervention). Effect sizes (Cohen’s *d*) ranged from .29 for social distance to .48 for pity.

## Discussion

This article demonstrates what photovoice can bring to health promotion practice, particularly focused on an application beyond the original exhibition. Here, we have focused on audience-awareness outcomes (compared with participant/artist outcomes), an aspect that is not often researched or evaluated. Our focus on the viewing audience is in keeping with the recommendation that photovoice projects use a social-ecological logic model to determine project processes and goals, including the individual, interpersonal, organizational, and community/societal levels (Strack et al., 2010). This photovoice application operated in the individual and interpersonal levels; the students developed individual and interpersonal awareness of stigma. At the time of the intervention (immediate outcomes), there was a small effect on personal stigma of the viewing audience, including significantly decreased perceptions of people with a mental illness as unpredictable and incompetent, decreased fear of people with a mental illness, and decreased desired social distance from people with a mental illness. At 1-month follow-up, there remained a significant reduction in students’ desire for social distance from people with a mental illness (indicating stigma reduction), although students also evidenced increased pity toward people with a mental illness that was not different immediately post-intervention (indicating an increase in stigma). Desire for social distance is a common stigma indicator, often linked to perceptions of dangerousness and discriminatory behavior (Corrigan et al., 2002) and so it is promising that the intervention improved stigma as measured by decreased social distance. However, the intervention also increased pity, which in this study was conceptualized as a stigmatizing response. The role of pity in stigma is complex—there is some evidence that pity can lead to helping behaviours, while there is other evidence that it can lead to negative outcomes such as paternalism and authoritarian responses (Fominaya et al., 2016). This study’s mixed results indicate the need to further clarify outcomes for this particular intervention and highlight the importance of evaluating outcomes for the purposes of health promotion practice to be assured that the intervention is having the desired effect.

The multimodal anti-stigma intervention preserved the first goal of photovoice that was articulated by Wang and Burris (1997): it enabled the Photovoice Artists to record and reflect their strengths and concerns, articulated as their shared experience of living with mental illnesses and their experience of recovery including barriers and enablers. The intervention also borrowed from the traditions of digital storytelling and photo elicitation. Photovoice shares many similarities with digital storytelling and photo elicitation: all are considered community-based participatory research methods, use images to convey community members’ experiences, and consider participant and community change in their processes. As with photovoice, digital storytelling can be used to encourage community building and advocacy regarding common issues (Gubrium, 2009; Gubrium et al., 2019). Photovoice uses photos to document daily realities to form the basis of group discussion, research, and advocacy (Wang and Burris, 1997) while digital storytelling is a methodology that guides participants to create short movies that tell stories about their experiences, and may or may not use a participant’s own photos and videos (Lambert, 2013). Photo elicitation is a qualitative methodology whereby photos are used to generate discussion and prompt inquiry (Glaw et al., 2017). As the focus of this study was on the application of the photos (which were conceptualized, discussed, developed, curated, and presented by persons with lived experience) for audiences, in this case health care students, and understanding the audience’s response, the method used in the present article represents an incarnation of all three traditions.

This article also demonstrates how photovoice is a good fit for multimodal anti-stigma interventions, articulating how photovoice methods and procedures align with stigma reduction best practices including social contact, multiple opportunities for social contact (e.g., through a live speaker, video, multiple first-voice speakers, etc.), education, and personal testimony (Knaak et al., 2014). In addition to being a form of parasocial contact (i.e., through photos, phototexts, and voiceovers), the photovoice images were used to frame and discuss issues; students were asked to select two images that made a personal impact (in terms of new knowledge, personal relevance, and/or emotional reaction), which was further discussed with the speaker and other audience members. Photovoice thus provides multiple opportunities for social contact—through the photos (in the present application, there were multiple first-voice speakers in the photovoice video) as well as through discussion with the photovoice participants themselves (personal testimony).

### Implications and Lessons Learned for Research and Practice

While this study provides support for a multimodal photovoice-based anti-stigma intervention, there are also several limitations to the study design and findings. First, this study used a small convenience sample of postsecondary students that consisted largely of White females studying nursing, which limits the generalizability of the findings. Additional research with larger samples are needed; larger samples would also permit controlling for variables that are known to be associated with stigma such as age, sex, and level of education. It is also possible that there was self-selection and dropout bias for participants in the study. In addition, there was no randomization to a control group, limiting our ability to attribute stigma change directly to the photovoice-based intervention. Similarly, we are unable to tease apart the impact of photovoice versus the other components of the multimodal intervention. A final limitation was the intervention’s broad focus on “mental illness” rather than a specific illness/disorder. While this approach is consistent with a large portion of stigma research, it fails to capture disorder-specific stigma, and it is possible that students conceptualized “mental illness” in different ways, which may than have differentially influenced their responses on the study measures. This limitation may also relate to the intervention’s mixed impact on stigma outcomes (i.e., improvement in social distance, worsening of pity). Stigma is a multifaceted construct and more research is needed to clarify the intervention’s impact on various stigma indicators.

This study demonstrated the application of photovoice to anti-stigma intervention for health care students, linking photovoice aims and procedures with best practices in stigma reduction. Personal stigma among health care providers and students presents a challenge for health promotion, and this study’s findings indicated that a multimodal photovoice-based anti-stigma intervention can improve personal stigma among health care students. Photovoice is also an empowerment opportunity for people with mental illnesses to play a role in educating the audience, using personal photos to convey their experiences living with mental illness, stigma, and recovery, and to engage health care students and providers in meaningful discussion—all toward the ultimate goal of reducing health care student and provider stigma. As such, future research should continue to investigate the utility of photovoice as a contact-based approach to reducing personal stigma in the viewing audience.
